# Examining Discursive Features in Crowdsourced Deliberation: Idea Generation through Elaboration

**DOI:** 10.1007/s10606-025-09535-z

**Published:** 2026-04-24

**Authors:** Tanja Aitamurto, Peter Royal, Jorge Saldivar

**Affiliations:** 1 Department of Communication, University of Illinois Chicago, 1007 W Harrison St, Chicago, IL 60607 USA; 2 Department of Computer Science, Pompeu Fabra University, Carrer de la Mercè, 12, Barcelona, 08002 Spain

**Keywords:** Civic technologies, Crowdsourced deliberation, Crowdsourcing, Deliberation, Democratic innovations, Policy-making, Disagreement, Participatory democracy

## Abstract

Civic technologies such as participatory budgeting and crowdsourced policymaking increasingly provide spaces for crowdsourced deliberation, an asynchronous, distributed, and self-selected form of deliberation. To understand the dynamics of crowdsourced deliberation, we examined deliberative quality, dis/agreement, and elaboration (rationale-sharing), and their association with idea generation in crowdsourced deliberation within a crowdsourced policymaking process led by a national government. The deliberative quality of the crowdsourced deliberation was high and positively associated with idea generation. Elaboration of perspectives was a key feature contributing to idea generation. We present considerations for the design of processes and platforms used in civic technologies to foster high deliberative quality, elaboration, and a balance of disagreement and agreement, which in turn can support idea generation in crowdsourced deliberation.

## Introduction

Policy deliberations increasingly occur online in virtual town halls, crowdsourced policymaking and participatory budgeting processes, and other participatory democracy applications (Johnson et al. 2020; Kim et al. 2019; Menendez-Blanco and Bjørn 2022). These civic technologies provide spaces for public deliberation, a fundamentally important building block of democracy (Cohen 1989; Gutmann and Thompson 1996; Habermas 1996). Deliberation enables citizens to express their opinions and ideas, provide rationales for their stances, and understand each other’s perspective (Bohman 1996; Gutmann and Thompson 1996; Habermas 1996). Traditionally, deliberation has been examined in political science as a structured, strongly facilitated, in-person exercise, often among pre-selected participants, in which the goal is to reach a consensus through constructive argumentation, and eventually, make a decision about the policy issue (Gutmann and Thompson 1996; Habermas 1996; Thompson 2008).

The digitalization of deliberative processes has contributed to the emergence of new forms of deliberation. One of these is crowdsourced deliberation, an asynchronous, distributed, and self-selected form of deliberation (Aitamurto and Landemore 2016; Aitamurto and Saldivar 2017a). Crowdsourced deliberation takes place within processes facilitated by civic technologies, such as participatory budgeting and crowdsourced policymaking. Crowdsourced deliberation seeks to enhance policy deliberations by uniting key elements from democratic deliberation (reasoned argument exchange characterized by respect and reciprocity among the participants) and idea crowdsourcing (gathering idea contributions from online crowds). Crowdsourced deliberation thus has a dual goal: fostering high-quality deliberation and generating policy proposals (ideas) (Aitamurto and Landemore 2016; Aitamurto and Saldivar 2017a).

Compared to traditional deliberative processes, crowdsourced deliberation shares the goal of fostering constructive argumentation but occurs online and is more loosely structured and less moderated and facilitated. Crowdsourced deliberation also has another key goal that derives from large-scale ideation processes: productive idea generation. In addition, crowdsourced deliberation is open for anyone to participate online, as opposed to the pre-selection of participants in more traditional forms of deliberation. With these features, crowdsourced deliberation is a type of a democratic innovation, which aims to engage citizens in democratic processes beyond elections (Smith 2009). Democratic innovations are perceived as potential remedies for the growing democratic deficit, which has permeated societies as citizens are less engaged with democratic processes (Norris 2011). One advantage of crowdsourced deliberation as a democratic innovation is that it can provide access to deliberative processes for a greater number of participants at low cost, whereas many other democratic innovations for deliberation, such as citizen juries and deliberative polls, traditionally suit only small-scale participation (Smith et al. 2009).

To better understand the dynamics of crowdsourced deliberation, in this paper we examine four intertwined key elements in digital policy deliberations at the discourse level: deliberative quality, disagreement, agreement, and elaboration. Prior literature on more traditional forms of deliberation shows that constructive deliberation can help participants learn about others’ perspectives and develop more reasoned opinions and compromises (Esterling et al. 2015; Price et al. 2002). For the benefits of deliberation to occur, the deliberation has to be of good quality: there should be respect and reciprocity in the process, and the participants should provide reasons for their positions (Bohman 1996; Gutmann and Thompson 1996; Fishkin 2009).

Disagreement is a double-edged sword in deliberation. On the one hand, constructive disagreement can enhance deliberation by contributing to the development of reasoned perspectives, new ideas, and compromises (Gutmann and Thompson 1996; Maia et al. 2021; Price et al. 2002). On the other hand, disagreement can also lead to opinion polarization in deliberation (Black and Wiederhold 2014; Esterling et al. 2015). In addition to disagreement, agreement is also an important element in deliberation as an ideal outcome (i.e., group consensus) (Cohen 1989; Gutmann and Thompson 1996; Habermas 1996). Discussants must reach agreement to some extent to develop compromises, and agreement helps to moderate disagreement so that it remains civil and productive (Maia et al. 2021; Stromer-Galley and Muhlberger 2009). Finally, elaboration is an essential element in constructive deliberation because it provides discussants with arguments by which they can assess the relative merits and drawbacks of policy proposals (Bohman 1996; Stromer-Galley 2007; Gutmann and Thompson 1996). Elaboration can take various forms—including rationales, definitions, examples, stories, and other explanatory or contextual information (Stromer-Galley 2007)—that can enhance deliberation by helping discussants understand each other’s perspective and assess policy proposals’ merits and drawbacks. When combined with (dis)agreement, elaboration, especially in the form of reason-giving or rationale sharing, can contribute to reasoned argumentation, a key element of deliberative quality (Bohman 1996; Stromer-Galley 2007; Gutmann and Thompson 1996).

By drawing on prior work on deliberation in both political science and computer-supported cooperative work (CSCW) and using participant interview, survey, and online comment data from an in-the-wild crowdsourced law reform process, we address the following research questions:

- **RQ1** What is the deliberative quality of crowdsourced deliberation?
- **RQ2** How is deliberative quality associated with new idea generation?
- **RQ3** What are the roles of disagreement, agreement, and elaboration in idea generation in crowdsourced deliberation?

These questions are particularly relevant and timely considering the growing body of work about civic technologies and democratic innovations, which facilitate crowdsourced deliberation, within the CSCW and broader HCI communities (Chen et al. 2019; Johnson et al. 2017; Kim et al. 2022; Palacin et al. 2019; Menendez-Blanco and Bjørn 2022). These communities are increasingly interested in designing and studying platforms and processes for civic engagement in applications for participatory democracy, such as civic technologies (Saldivar et al. 2019; Corbett and Le Dantec 2021; Taylor et al. 2018; Boehner and DiSalvo 2016; Walker 2018), deliberation and argumentation technologies (Johnson et al. 2017; Chen et al. 2019; Palacin et al. 2019; Kim et al. 2022), and crowdsourcing for civic purposes (Mahyar et al. 2018; Lee et al. 2018). The goals of these processes and technologies include ideation and deliberation with constructive argumentation.

These are the key findings:

- The quality of the crowdsourced deliberation was high despite its differences from more traditional forms of deliberation.
- The type of disagreement mattered for idea generation: elaborated disagreement was the most conducive to idea generation.
- The presence of both elaborated disagreement and elaborated agreement was conducive to idea generation.
- Reason-giving, a form of elaboration, had an especially strong association with idea generation.
- Fostering elaboration, and reason-giving in particular, should be prioritized in the design of processes and technologies for facilitating crowdsourced deliberation. Furthermore, disagreement should not be regarded solely as a negative element in deliberation because constructive disagreement can provide fertile ground for idea generation.

These findings provide knowledge about the factors affecting ideation and argumentation in crowdsourced deliberation, thus contributing to more effective designs for civic technologies and other applications of participatory democracy, which strive for both ideation and deliberation. In a broader context, the findings help us understand what conditions are conducive to constructive deliberation on a large scale and, consequently, how to leverage civic technologies for democratic deliberation and constructive ideation and thereby promote greater democratic participation and citizen engagement.

The paper is structured as follows. In the 2 section, we first review the roles of crowdsourcing and deliberation in policymaking as well as the distinctive features of crowdsourced deliberation that differentiate it from more traditional forms of deliberation. We then discuss the roles of dis/agreement, elaboration, and other discursive features of crowdsourced deliberation as they relate to deliberative quality and idea generation. In the 3 section, we introduce the case profile—the crowdsourced Association Act reform in Finland—in more detail. We then describe the data gathering and analysis methods pertaining to each research question. In the 4 section, we present the results pertaining to RQ1–3, respectively. In the 6 section, we discuss the findings according to the framework of the dual goals of crowdsourced deliberation (ideation and constructive deliberation), followed by design considerations and directions for future work. The 7 summarizes the main findings.

## Key Concepts and Related Work

### Crowdsourced Deliberation through Democratic Innovations

Crowdsourced deliberation derives from traditional forms of democratic deliberation, which entails the fair and equal consideration of ideas and exchanges of well-reasoned arguments (Habermas 1984; Cohen 1989). Democratic deliberation has traditionally taken place in democratic processes such as parliamentary debates, jury deliberations, and in-person town hall meetings (Fishkin 2009). With the digitalization of democratic processes, many of these deliberations have moved online. As a parallel development, digital democratic innovations have created new spaces for deliberation. Democratic innovations are processes and technologies designed to enhance democratic participation and engage citizens in democratic processes beyond elections (Smith 2009). Participatory budgeting, crowdsourced policymaking, and other civic technologies are commonly examined democratic innovations (Kim et al. 2019; Menendez-Blanco and Bjørn 2022; Saldivar et al. 2019). These participatory democracy applications can help to incorporate non-experts, local knowledge, and new perspectives into the policymaking process and thus contribute to democratic values in society, namely, inclusiveness, transparency, and collaboration (Prpić et al. 2015; Aitamurto and Landemore 2016, 2015; Landemore 2017).

Many of these democratic innovations and civic technologies provide spaces for crowdsourced deliberation, a type of online deliberation that is distributed, asynchronous, and depersonalized and therefore takes place at the “deliberative systems” level (Aitamurto and Landemore 2016; Parkinson and Mansbridge 2012). Crowdsourced deliberation has been theorized to generate new ideas, refine existing or emerging ideas, and yield better solutions to policy issues by recombining aspects of different proposals (Landemore and Page 2015; Aitamurto and Landemore 2016; Landemore 2020). A government typically initiates and oversees crowdsourced deliberation as one component of a larger process involving democratic innovations, which distinguishes crowdsourced deliberation from online deliberation in other fora, such as newspapers’ comments sections or Reddit. Governments use crowdsourcing to achieve two primary goals: (1) to develop better policies by leveraging citizens’ knowledge and insights and (2) to promote civic engagement (Aitamurto and Chen 2017). However, even when crowdsourcing is deployed in a policy reform process, elected representatives, such as members of parliament, still decide the goals of the crowdsourcing process and how to use its outcomes. Crowdsourced deliberation is therefore a method for participatory democracy—in which the goal is to engage citizens in political processes between elections (Pateman 1970)—rather than for direct democracy (Frey 1994) because the participants do not have decision-making power over the policy. On the ladder of citizen participation (Arnstein 1969), crowdsourced deliberation falls on the “consultation" level, several steps removed from full citizen control. However, crowdsourcing can help to incorporate non-experts, local knowledge, and new perspectives into the policymaking process and arguably has the potential to make policymaking more inclusive, transparent, and collaborative (Prpić et al. 2015; Aitamurto and Landemore 2016, 2015; Landemore 2017).

**Table 1:** Comparison of the key features of crowdsourced deliberation and traditional democratic deliberation

*Feature*	Crowdsourced deliberation	Traditional democratic deliberation
*Goals*	Generation of policy proposals (ideas); constructive argumentation in a high-quality deliberation	Consensus through constructive argumentation in a high-quality deliberation
*Locus of control*	Crowdsourcer	Organizer of the deliberation
*Scale*	Large	Small
*Participant sample*	Self-selected	Random; representative
*Facilitation*	Often unstructured	Structured; systematic; time allocated equally among discussants to support deliberative quality
*Communication standards*	Rationality, civility, equality, constructive tone	Rationality, civility, equality, constructive tone
*Information*	Prompts for idea generation, possibly accompanied by additional information	Delivered by materials and experts (also live talks by experts, e.g., in deliberative polls)
*Ideal societal benefits*	Civic engagement; understanding others’ perspectives; harnessing the crowd’s creativity and knowledge for policy proposals	Civic engagement; understanding others’ perspectives; consensus
*Location*	Online platform	Traditionally in-person

Both crowdsourced deliberation and traditional democratic deliberation, when taking place online, are types of online deliberation, which is an umbrella term for various types of deliberations taking place online. Online deliberation as a concept may thus refer to structured deliberations similar to crowdsourced deliberation, or more free-from online discussions, which may take place on social media platforms, online discussion forums and in newspapers’ comment sections without specific structure and goals.

While crowdsourced deliberation shares many features with traditional democratic deliberation, there are also several fundamental differences between these two forms of deliberation. Table 1 summarizes the key similarities and differences between crowdsourced deliberation and traditional democratic deliberation.

First, crowdsourced deliberation differs from more traditional forms of democratic deliberation in terms of its *goals*. In addition to constructive argumentation, crowdsourced deliberation aims to gather diverse knowledge and generate ideas, goals adopted from large-scale idea crowdsourcing processes, while traditional democratic deliberation strives for consensus through constructive argumentation.

Another important property of the types of deliberations is the *locus of control.* In crowdsourced deliberation, the locus of control is within the crowdsourcer, which in policy deliberations often is a governmental body or a non-profit organization. The crowdsourcer determines the design and execution of the crowdsourced deliberation process, including the topic of deliberation, time, location, and the method for processing the crowd’s input and managing the outcome of the crowdsourced deliberation. Similarly, in traditional democratic deliberation, it is the organizer of the deliberation that holds the control over the process and the output.

*Scale* is another property that can differ between crowdsourced deliberation and traditional democratic deliberation. Unlike traditional democratic deliberation, which is typically organized in in-person roundtable discussions in small groups of participants, crowd-sourced deliberation leverages online platforms to accommodate a much larger number of participants, potentially allowing policymakers to gather a larger and more diverse set of policy proposals. Another important advantage of the large scale of crowdsourced deliberation is the expanded capacity for participation relative to more traditional forms of deliberation, greatly increasing the number of citizens who can participate in a deliberative process. The potential to engage a larger group of participants at low cost and to gather diverse knowledge and insights makes crowdsourced deliberation an appealing civic engagement method for governments.

Regarding the *participant sample*, self-selection is another key factor differentiating crowdsourced deliberation from several other participatory democracy methods involving more traditional forms of deliberation, such as deliberative polls (Fishkin 2009) and citizens’ assemblies (Lacelle-Webster and Warren 2021), in which organizers select participants via random or quota sampling, thus forming a “mini-public,” which is considered to represent a larger public. The mini-public approach in sampling has been called “the gold standard” of sampling in public deliberation (Mansbridge 2010) and is generally the sampling method deployed in traditional democratic deliberation processes. Because participants in crowdsourced deliberation are self-selected, they are unlikely to constitute a representative sample of a larger population, so the results of a crowdsourced deliberation cannot serve as a proxy for “public opinion.” In crowdsourced deliberation, the goal is not to generate a “mini-public” to represent a larger public but rather to find relevant knowledge and generate ideas in the form of policy proposals. These distinctive features of crowdsourced deliberation affect the processing and usage of the crowd’s input. The policymakers overseeing a crowdsourced deliberation process typically synthesize the crowd’s contributions into a summary, which serves as one data point among many that policymakers consider (Aitamurto and Landemore 2016).

Another key difference between crowdsourced deliberation and traditional democratic deliberation is the role of *facilitation* or moderation. In traditional democratic deliberation settings, trained facilitators intervene to ensure that deliberation adheres to normative ideals and established guidelines as closely as possible, including respect, reasoned argumentation, and balanced participation (Friess and Eilders 2015). This entails ensuring that participants have roughly equal opportunity and time to contribute. In crowdsourced deliberation, in contrast, the participants deliberate asynchronously while proposing ideas for improving policy in a relatively free-form process, typically with very little, if any, facilitation (Aitamurto and Landemore 2016; Prpić et al. 2015). The organizers of a crowdsourced deliberation may either pre- or post-moderate but do not necessarily intervene in or guide the deliberation as it occurs.

For a communicative act to qualify as democratic deliberation, the interaction must meet certain conditions and, particularly, *communication standards* such as rationality, civility, equality, and a constructive tone (Friess and Eilders 2015). These same standards apply to crowdsourced deliberation. The expectation for these standards differentiates crowdsourced deliberation and traditional democratic deliberation from various online discussions, which may occur on social media platforms and newspapers’ comment sections without the expectation for rationality, civility, equality, and a constructive tone.

Procedures for providing and accessing *information* also differ between crowdsourced deliberation and traditional democratic deliberation. Due to the complexity of policymaking deliberations, it is important for discussants to have sufficient information to debate in an informed and rational manner (Friess and Eilders 2015; Smith et al. 2009). Structured information on the deliberation topic is often provided in democratic deliberations (Luskin et al. 2006; Min 2007; Muhlberger 2005), sometimes including the opportunity to watch experts debate about the topic (Strandberg and Grönlund 2012) or submit questions to an expert panel (Fishkin 2009; Luskin et al. 2006). In contrast, in crowdsourced deliberation, information is typically provided to the participants on the online platform. The organizers do not control whether the participants have read the materials. The participants may exchange information with each other and learn from materials provided on the online platform, but facilitators typically do not intervene in a structured or systematic manner to foster these behaviors (Aitamurto and Landemore 2016; Prpić et al. 2015).

Regarding the *location* of deliberation, democratic deliberation has traditionally taken place in person,^1^ whereas crowdsourced deliberation takes place on online platforms, which are open for the public to participate. The platforms on which crowdsourced deliberation occurs are often designed for *either* idea generation, on the one hand, *or* deliberation and argumentation, on the other, rather than integrating features for both of these purposes (Aitamurto and Landemore 2016, 2015; Aitamurto and Chen 2017; Iandoli et al. 2018). Ideation platforms such as IdeaScale, IdeaPlace, Howspace, and IdeaHound (Siangliulue et al. 2016) are designed primarily for gathering a large number of ideas rather than for soliciting justifications for ideas, i.e., prompting participants to provide reasons and evidence to support their assertions. In contrast, deliberation and argumentation tools, such as Consider.it (Kriplean et al. 2012) and Reflect (Kriplean et al. 2012), are designed to facilitate deliberation and consensus building by prompting participants to provide rationales for their opinions and proposals, thus supporting the goals of traditional democratic deliberation initiatives (Quinto et al. 2021; Klein 2011). Table 2 compares the key goals and features of some of the tools and platforms that governments use in their civic engagement initiatives. The table summarizes the key features for supporting deliberation and/or ideation and shows that they are often designed for either deliberation or ideation rather than both.

**Table 2:** Comparison of platforms and tools for deliberation and ideation

Platform / Tool	Primary goal	Specific features supporting argumentation	Specific features supporting ideation
Consider.it (Kriplean et al. 2012)	Deliberation	Gathering pro/con statements; exploration of aggregated positions	None
DebateBot (Kim et al. 2021)	Deliberation	Chatbot for eliciting justifications and reaching consensus	None
DebateHub (Quinto et al. 2021)	Deliberation	Pro/con argumentation; aggregation and visualization of arguments	None
Decidim$^{1}$ (Aragón et al. 2017)	Deliberation	Collaborative proposal creation tool; commenting on proposals	None
LiquidFeedback (Behrens et al. 2014)	Deliberation	Issue-based discussion; formal proposal submission; feedback and iteration	None
MIT Deliberatorium (Klein 2011)	Deliberation	Argument mapping (tree structure) and visualization	None
MOOD (Massive Open Online Deliberation; Verdiesen et al. 2016)	Deliberation	Pro/con argumentation; measuring moral acceptability and social acceptance of alternatives	None
Nudging (Menon et al. 2020)	Deliberation	Word-count anchors, partitioned text fields (position, explanation, evidence), and reply choice prompts	None
Reflect (Kriplean et al. 2012)	Deliberation	Restating positions	None
SolutionChat (Lee et al. 2020)	Deliberation	Visualizes discussion stages and featured opinions; recommends contextually appropriate moderator messages	None
HowSpace$^{2}$	Ideation	None	Prompts for ideas
IdeaHound (Siangliulue et al. 2016)	Ideation	None	Prompts for ideas; analyzing semantic relationships among ideas
IdeaPlace (formerly Spigit)$^{3}$	Ideation	None	Prompts for ideas
IdeaScale$^{4}$	Ideation	None	Prompts for ideas

$^{1}$See: https://decidim.org/features

$^{2}$See: https://howspace.com

$^{3}$See: https://www.planview.com/products-solutions/products/ideaplace

$^{4}$See: https://ideascale.com/product-tour

### Deliberative Quality and Ideation in Crowdsourced Deliberation

In democratic deliberation, ideally, participants exhibit mutual respect, reciprocity, and civility and justify their stances, features that serve as standards for democratic deliberation and indicate deliberative quality (Black and Wiederhold 2014; Gutmann and Thompson 1996). The higher the quality of deliberation, the more likely it is to yield benefits for participants, such as learning and understanding others’ viewpoints (Gutmann and Thompson 1996; Parkinson and Mansbridge 2012; Landemore and Page 2015; Esterling et al. 2015).

Deliberative quality has been measured in traditional democratic deliberation settings (Fishkin 2009; Mansbridge 2010; Stromer-Galley 2007), while the quality of crowdsourced deliberation remains understudied. It is especially important to examine the deliberative quality of these unfacilitated, self-selected, asynchronous online deliberations as democratic innovations and civic technologies are becoming more common. Examining deliberative quality will inform us whether crowdsourced deliberation can be a space for high-quality deliberation and constructive argumentation and thus help to fulfill democratic values in society. Therefore, we pose the following research question:

- **RQ1** What is the deliberative quality of crowdsourced deliberation?

To address this question, we used data from a crowdsourced deliberation led by the Ministry of Justice of the government of Finland. We used survey and interview data to gauge the participants’ perceptions of the deliberative quality of the crowdsourced deliberation. We also developed a coding scheme (described in section 3.2.1) to analyze online comment data from the crowsourced deliberation, using the following discursive features as indicators of deliberative quality:

- Elaboration is a component of reasoned opinion expression that accompanies an expression of (dis)agreement with a proposal or another opinion. It can take various forms, including giving reasons or presenting evidence to justify one’s opinion. Elaboration helps to differentiate a simple expression of (dis)agreement (i.e., “I [dis]agree”) from one that includes some further detail that would enable other discussants to understand and respond to it (Stromer-Galley 2007).
  - *Giving reasons* or sharing a rationale for one’s stance provides other discussants with material on which to base rational and constructive disagreement and/or agreement (Stromer-Galley 2007; Gutmann and Thompson 1996).
  - *Presenting evidence* provides material—such as books or government reports—for other discussants to consider, which can improve the ideas that emerge from deliberations (Stromer-Galley 2007).
  - *Clarifying a position* on an issue provides other participants with material for discussion and helps participants understand each other’s ideas and opinions.
- Participants can also *provide information*, making comments that convey information in a neutral/nonpartisan manner. In other words, providing information is analogous to elaboration but does not accompany opinion expression (i.e., [dis]agreement with a proposal or another opinion).
- *Asking genuine questions* that sincerely seek information from others signals engagement (Stromer-Galley 2007).

Other discursive features of deliberation describe how a comment functions in relation to others rather than its content. These features serve as indicators of interactivity, reciprocity, and civility and can thus indicate deliberative quality:

- *Responding to other participants’ comments* and *staying on topic* reflect reciprocity, engagement, and active listening (Gutmann and Thompson 1996; Stromer-Galley 2007).
- *Staying on topic* shows that the participants in the deliberation can focus on the topic at hand (Stromer-Galley 2007).
- A *constructive tone* shows respect towards others, which is an important factor contributing to the quality of deliberation (Gutmann and Thompson 1996; Stromer-Galley 2007).

While some prior work shows that crowdsourced deliberation can satisfy many requirements of democratic deliberation (Aitamurto and Landemore 2016)—for example, that participants exchange arguments in a free, equal, and inclusive manner—we still know very little about crowdsourced deliberation and its dynamics. It is especially important to examine discursive features that may affect ideation, a key goal of crowdsourced deliberation alongside constructive argumentation. The more productive the ideation is, the more likely it is that the crowd will share valuable knowledge. Studies of ideation and innovation processes have shown that discursive features such as as reciprocity and sharing justifications can contribute to productive ideation (No et al. 2017; Xiao 2014, 2011, 2013). These discursive features may also play an important role in crowdsourced deliberation, yet this question remains understudied. To better understand the role of deliberative quality in idea generation in crowdsourced deliberation, we posed the following research question:

- **RQ2** How is deliberative quality associated with new idea generation?

To address this question, we applied our coding scheme to the online comment data from the crowdsourced deliberation, focusing on discursive features indicating deliberative quality and the number of ideas presented in comments. We developed a multivariate linear regression model based on our theoretical framework and the coding results. In this model, we used the same discursive features as in RQ1: *giving reasons*, *presenting evidence*, *clarifying a position*, *providing information*, *asking genuine questions*, *responding to other participants’ comments*, *staying on topic*, and *constructive tone*.

### Disagreement, Agreement, and Elaboration in Deliberation

Both disagreement and agreement play important roles in deliberation (Stromer-Galley 2007; Stromer-Galley and Muhlberger 2009). In deliberation, disagreement occurs when discussants express a perceived difference of opinion, values, or beliefs (Stromer-Galley 2007). Deliberation implies an expectation of disagreement because deliberation requires multiple perspectives and a lack of consensus (Stromer-Galley et al. 2015; Esterling et al. 2015; Gutmann and Thompson 1996). By eliciting rationales and evidence, disagreement can contribute to participants’ ability to generate reasons for their own and others’ opinions and the development of mutual understanding among discussants (Price et al. 2002; Gutmann and Thompson 1996; Maia et al. 2021). Disagreement has also been found to contribute to new idea generation in crowdsourced, large-scale ideation processes (No et al. 2017). Disagreement is more likely to be beneficial when it is expressed in a civil manner and accompanied by elaboration (e.g., when discussants share their rationales for their arguments, ideas, and claims) (Black and Wiederhold 2014; Gutmann and Thompson 1996; Maia et al. 2021). Furthermore, expressions of agreement help discussants establish common ground, mutual respect, and a willingness to compromise in policy deliberations and group problem-solving. Expressions of agreement that accompany expressions of disagreement can mitigate the potential for disagreement to be perceived as impolite, thus helping to maintain civility and mutual respect despite differences of opinion (Stromer-Galley 2007; Stromer-Galley and Muhlberger 2009; Maia et al. 2021).

Elaboration is an essential element of both disagreement and agreement in deliberative processes because it provides discussants with material by which they can judge the merits of and potential justifications for a policy proposal (Habermas 1984; Gutmann and Thompson 1996; Stromer-Galley 2007). An elaboration is a statement with additional argumentation or information, such as a justification, a definition, or an example (Stromer-Galley 2007). Elaboration in the form of rationale sharing has also been identified as important in ideation processes in small group collaboration because elaboration helps build common ground and a shared mental model (No et al. 2017; Paulus et al. 2019; Xiao 2014). In a similar vein, research on ideation and innovation processes has shown that awareness of other discussants’ reasoning during ideation helps group members generate more ideas (No et al. 2017; Xiao 2011, 2013). Elaboration red, especially in the form of reason-giving or rationale sharing, thus not only helps deliberation fulfill its democratic potential but can also support idea generation, another key goal of crowdsourced deliberation. In idea crowdsourcing, participants’ exposure to each other’s rationales can help them establish common ground, raise awareness of each other’s knowledge, and improve the average quality of ideas (Xiao 2014, 2013). Thus, in crowdsourced deliberation, elaboration supports deliberative democratic ideals of communicative inclusion and reasoned argumentation as well as idea generation.

Taken together, disagreement, agreement, and elaboration are central elements in deliberation (Gutmann and Thompson 1996; Stromer-Galley 2007) and have also been identified as factors that can contribute to ideation in innovation processes (Iandoli et al. 2018; Paulus et al. 2019; Xiao 2014). These discursive features of deliberation can contribute to new idea generation, improve the quality of generated ideas, enhance understanding of problems, and help to refine ideas by prompting rational justification (Iandoli et al. 2018; No et al. 2017; Xiao 2014). However, the roles of disagreement, agreement, and elaboration in ideation in crowdsourced deliberation remain understudied. Knowing more about the roles of dis/agreement and elaboration in idea generation can help us understand the dynamics of crowdsourced deliberation and improve the design of processes and technologies for more productive crowdsourced deliberation within civic technologies and democratic innovations. To examine the relationship between dis/agreement, elaboration, and idea generation in crowdsourced deliberation, we posed the following research question:

- **RQ3** What are the roles of disagreement, agreement, and elaboration in idea generation in crowdsourced deliberation?

To address this question, we developed a coding scheme (described in section 3.2.1) to analyze online comment data from a crowdsourced deliberation led by the Ministry of Justice in the government of Finland. We then further analyzed the data using a multivariate linear regression model and a multi-step inferential analysis, which we describe in more detail in the next section.

**Fig. 1:**
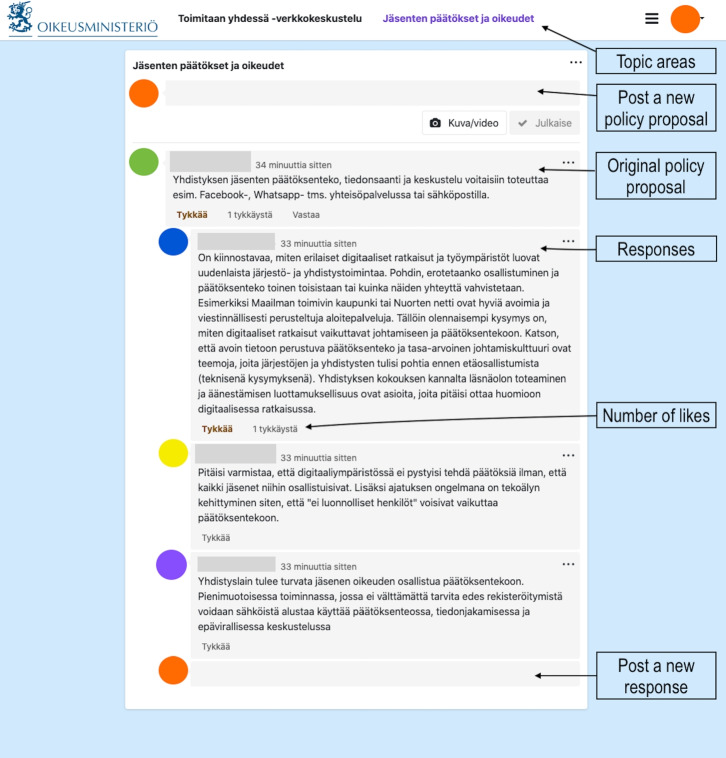
The user interface of the platform, on which the crowdsourced deliberation took place. The participant names and profile pictures are blurred

## Case Profile, Methods, and Data

In this section, we describe the profile of the case study—the crowdsourced Association Act reform in Finland—and the data gathering and analysis methods. We first provide contextual details about the policy reform process and then review the data gathering and analysis methodology.

### Crowdsourced Association Act Reform

Finland’s Ministry of Justice launched the crowdsourced deliberation process this study examines in May 2019. The Ministry sought to crowdsource ideas for reforming a law called the Association Act (26.5.1989/503), which governs established forms of civic engagement, namely, the establishment and management of associations. The crowdsourced deliberation took place on a crowdsourcing platform called HowSpace (www.howspace.com), which was open for anyone to participate. The participants could access the platform by signing up with an email address, and they could choose to remain anonymous or use their real names on the platform. Both of these options were used by the participants. The platform was provided in three languages: Finnish, Swedish, and English. Three representatives of the Ministry of Justice participated in the deliberation by posting policy reform proposals as solutions to policy issues and asking and answering questions. The representatives stated that they did not remove any comments in post-moderation. The Ministry announced the crowdsourcing initiative through various digital channels, including social media and a website dedicated to the Association Act reform. In addition to online crowdsourcing, the Ministry of Justice arranged a series of workshops in cities around Finland in May and June 2019 to discuss the legislative reform with citizens. The Ministry of Justice representatives gathered policy proposals from the workshop participants and posted the proposals in the crowdsourced deliberation process, alongside with other policy proposals.

The Ministry formulated three main topic areas for deliberation on the crowdsourcing platform. Participants were prompted to either post their own ideas for improving the law or react to proposals developed by the Ministry representatives. The participants could post their ideas, reply to existing posts, and like others’ proposals or comments. Figure 1 shows the user interface, on which the deliberation took place. The platform represented a large-scale idea crowdsourcing technology rather than a deliberation technology because there were no specific features for mapping arguments or synthesizing pros and cons of proposals. The Ministry chose the platform for pragmatic reasons—including cost, accessibility, and that Howspace is a local Finnish company—rather than selecting a platform specifically designed to foster deliberative quality.

The crowdsourced deliberation lasted four weeks in June 2019. 151 participants took part, and 488 comments were submitted on the platform. When the crowdsourcing process had concluded, the Ministry representatives analyzed the crowd’s input and published the results in a report. The legislative reform concluded in 2022, and the reformed law went into effect in February 2023.

#### Participant Profile

We distributed an online survey to the registered participants, 52 of whom completed the survey, resulting in a 30% response rate, which is consistent with online survey response rates in general (Shih and Fan 2008). By responding to the survey, the participants were entered into a raffle for a gift card. The vast majority of the survey respondents (90%) were middle-aged (35–54 years) or older. A slight majority (54%) were male. Most of the the respondents were working full-time (68%). The participants were highly educated: 73% of the respondents had completed high school, nearly half (46%) had a Master’s degree, and 19% had an institute-level vocational degree. About one-fifth (22%) of the respondents worked in associations as staff members. The respondents were active participants and volunteers in associations: one-fifth (21%) participated in an association as a hobby. The participant profile, based on the survey responses, thus reflects the self-selected nature of crowdsourced deliberation: those who are interested in the subject participate, as opposed to a randomly selected participant sample.

### Methods and Data

In this study, we examined the following three research questions:

- RQ1 What is the deliberative quality of crowdsourced deliberation?
- RQ2 How is deliberative quality associated with new idea generation?
- RQ3 What are the roles of disagreement, agreement, and elaboration in idea generation in crowdsourced deliberation?

We used several data gathering and analysis methods to address our research questions. This multimethod approach (Brewer and Hunter 1989) enabled us to examine our research questions from multiple angles. In the following, we review the methodology per each research question.

To address RQ1 about the deliberative quality of crowdsourced deliberation, we first developed a coding scheme to analyze the online comment data and then used inferential statistics to analyze the online comment data further. We also used qualitative methods to analyze the participant survey, interview, and online comment data and descriptive statistics to analyze the survey data to address RQ1.

To address RQ2 about the association between deliberative quality and new idea generation, we used the coding scheme to analyze the online comment data and then deployed inferential statistics to analyze the online comment data further. We also used descriptive statistics to analyze the survey data to address RQ2.

To address RQ3 about the roles of disagreement, agreement, and elaboration in idea generation in crowdsourced deliberation we used the coding scheme to analyze the online comment data.

This study was approved by the Institutional Review Board. In the following, we describe the data gathering and analysis methods in more detail.

#### Crowdsourced Comments and Coding Scheme

The comment data (488 comments) were exported from the platform and translated into English. To analyze the data, we developed a coding scheme based on studies of crowdsourcing (Aitamurto and Landemore 2016, 2015), deliberation (Landemore and Page 2015; Fishkin 2009), and dis/agreement (Black and Wiederhold 2014; Stromer-Galley and Muhlberger 2009; Stromer-Galley 2007) see section 2.2). The categories in the coding scheme are described in Table 3, with definitions and examples for each code.

The coding scheme includes codes for opinion expression—*agreement* and *disagreement*—and indicators of deliberative quality. Indicators of deliberative quality include content-based features (i.e., features of participants’ use of language) and features describing a comment’s relationship with other comments, including *responding to another participant’s comment* and *staying on topic*. Content-based features include *elaboration*, which encompasses behaviors such as *giving reasons*, *presenting evidence*, and *clarifying a position*. Additional content-based indicators of deliberative quality include *providing information*, *asking genuine questions*, and a *constructive tone* (i.e., a tone characterized by politeness and respect towards other participants). The codes *elaboration*, *gives reason(s)*, *presents evidence*, and *clarifies position* were applied only to comments that expressed *(dis)agreement*. The code *provides information* is analogous to elaboration but does not accompany an expression of *(dis)agreement*. Apart from these conditions, multiple codes could apply to the same comment.

We use Stromer-Galley’s (2007) definition of elaboration to differentiate between simple (dis)agreement (e.g., “I (dis)agree”) and elaborated (dis)agreement before coding for specific forms of elaboration (e.g., reason-giving, presenting evidence). Elaboration is a relatively broad category of discursive behaviors within deliberation that go beyond merely expressing agreement or disagreement, thus providing some discursive material for other participants to consider and respond to. Some form of elaboration is necessary for deliberation to progress beyond simple disagreement or agreement. In accordance with Stromer-Galley’s (2007) definition, we regard elaboration as a component of reasoned opinion expression and therefore a potential indicator of deliberative quality.

We applied the initial coding scheme to a sample of 150 comments, which represented 30% of the total number of comments. Three researchers first coded the pilot data individually. We then discussed any discrepancies. To resolve discrepancies, we consulted the literature, discussed the best approach to address our research question, and refined the coding scheme accordingly. We repeated this revision process until we reached consensus on the final version of the scheme.

Two researchers then coded each comment for the presence or absence of *disagreement*, *agreement*, *elaboration*, and at least one *new idea*. We also counted the *number of ideas* presented in each comment. Table 3 provides a definition and examples for each coding category. We used Krippendorff’s alpha to check intercoder reliability between the two coders. We report the *k*.*alpha* for each code in Table 3. Table 5 (see section 4.1.2) shows the frequencies of discursive features indicating deliberative quality in the comment data; Table 7 (see section 4.2.1) shows the frequencies of disagreement, agreement, elaboration, and new ideas.

In the comment data analysis, we included posts by the discussion facilitators (three representatives of Finland’s Ministry of Justice), which constituted about 25% of the total number of comments. We decided to do so because of the role of the Ministry representatives in this crowdsourced deliberation: They participated to the deliberation in a manner similar to the participants rather than as supervisors or moderators. As shown in the comparative tables of the participants’ and the facilitators’ comments in the Appendix (section A), the discursive features occurred with similar frequencies in the facilitators’ comments compared with the participants’ comments, with the main differences being that the facilitators’ comments more often provided information and evidence while they less frequently expressed opinions and reasoning, reflecting their role as information providers.

**Table 3:** Description of the coding scheme: Code categories, values, definitions, and examples

Name of code	Definition	Example
*Disagreement, Agreement, Elaboration*		
Disagreement (values: 0/1; IR=0.89)	When the participant expressed disagreement with a proposal or idea.	“I do not support the proposal.” (simple disagreement) “I think it’s a bad solution, because nobody remembers this sort of distinction in the daily practice of association activities.” (elaborated disagreement) “I support the proposal.” (simple agreement) “I support this. Openness is always a good thing, and when there’s a provision on it, members have something they can invoke.” (elaborated agreement)
Agreement (values: 0/1; IR=0.89)	When a participant signaled support for another participant’s or moderator’s comment.	
Elaboration (values: 0/1; IR=0.95)	Any expression of disagreement or agreement was coded as either simple or elaborated; an expression of dis/agreement was categorized as simple when it expressed dis/agreement without any elaboration and as elaborated according to the definition by Stromer-Galley (2007, p. 10).	
*Idea Generation*		
New idea (values: 0/1; IR=0.95)	Comment contributed at least one new idea to the discussion.	“I think it would be a good idea to allow online meetings, and mention that in the law for clarity’s sake, but leave the actual issue to the rules of the association. Then those associations that believe that their members would have an equal opportunity to participate could change their rules.”
Number of ideas (values: numeric)	The number of distinct ideas was counted for each comment.	“There should be several founders so that there would be more than one person monitoring the activities, already because of potential abuses.” (number of ideas=1)

Name of code	Definition	Example
*Deliberative Quality*		
Gives reason(s) (values: 0/1; IR=0.92)	Comment included a phrase or statement justifying or supporting the commenter’s claim(s); only applied to comments featuring disagreement and/or agreement.	“I think equality is an idealistic principle that is strived towards but cannot in any way be achieved in practice, since people’s own limitations, skills, and geographical differences cannot ever be fully compensated, no matter what the law says.”
Presents evidence (values: 0/1; IR=0.94)	Comment presented some kind of evidence to support the presented claim(s); only applied to comments coded as featuring disagreement and/or agreement.	“[T]he level of costs can be influenced by the level of costs of the identification service investment and of implementing the service: for example, according to the World Bank, a serviceable trade register system can be bought for a couple of hundred thousand euros, and the service may be highly automated, which differs considerably from the cost level of the (cost-plus pricing based) Register of Associations system.”
Asks question(s) (values: 0/1; IR=0.95)	Comment included a genuine question according to Stromer-Galley (2007, p. 12).	“Does the current Associations Act prevent the creation of the role mentioned above? If there have been difficulties, it might be necessary to clarify the matter.”
Provides information (values: 0/1; IR=0.92)	Comment did not indicate a particular position on an idea and provided information (e.g., clarifying, factual, contextual).	“Good point. For example, the Foundations Act (Chapter 3, section 3, subsection 5) provides that if the executive committee decision is made without holding a meeting, the decision must be recorded, signed, numbered and stored in the same way as the law provides on the committee minutes of a ’traditional meeting.’ ”
Clarifies position (values: 0/1; IR=0.89)	Comment clarified or elaborated on a position on an idea, typically in response to another comment, such as a challenging question.	*Commenter 1*: “How would it make the activities of small associations more difficult, and could this somehow be avoided even if disqualification regulations were changed?” *Commenter 2*: “A concrete example: The association’s members are a married couple and one other person. One of the couple is the president. You are making a decision on an issue which disqualifies the married couple. You can’t make any decision, since there is no qualified majority.”

Name of code	Definition	Example
Acknowledges problem (values: 0/1; IR=0.89)	The commenter explicitly acknowledged the existence and/or legitimacy/validity of a problem mentioned in another comment.	*Commenter 1*: “When allowing meetings to take place only online, it’s also necessary to think of a fallback procedure for when the internet connection isn’t working.” *Commenter 2*: “This is an important issue, since power cuts can be quite long, and it might even be several days before an online meeting can be held.”
Topic shift (values: 0/1; IR=0.93)	Comment changed the subject of discussion relative to the preceding comment(s).	*Commenter 1*: “The starting point for access to information could be complete openness for all members.” *Commenter 2*: “I support openness, but how does it happen in practice. All members are never in the same place at the same time.” *Commenter 3*: “The starting point for representation could be the right of every member to conclude agreements or otherwise represent the group.”
Responds to previous comment (values: 0/1; IR: 0.95)	Comment responded directly to a previous comment, signified by, e.g., explicitly naming a previous commenter or referencing the previous comment.	*Commenter 1*: “With online meetings, another problem is the reliable identification of participants: are the people on the other side of the internet really who they claim to be... It’s too easy to pretend to be someone else, making it possible to manipulate the association meeting.” *Commenter 2*: “It’s perfectly possible and works well. In education (e.g. vocational training), online platforms are already being used, with students participating in teaching remotely. A good platform for meetings and negotiations is e.g. Skype, which is free, and through which even calls are free. Many things just require people to adapt to new things.”
Constructive tone (values: 0/1; IR=0.89)	Comment expressed claims, opinions, etc. in a civil, constructive manner; this meant that the comment did not have an aggressive, hostile, or contentious tone but instead engaged other participants in a polite manner.	“In my opinion, association members should accept not only the rules of the association, but also its values. Violating the values of an association can be comparable to a rule violation.”

#### Multivariate Linear Regression Model for Online Comment Data Analysis

To examine the relationship between deliberative quality and idea generation (RQ2) as well as the relationships between dis/agreement and idea generation and between elaboration and idea generation (RQ3), we developed two multivariate linear regression models. In these models, an online comment was the unit of analysis, and 488 comments were analyzed.

In the model addressing RQ2, deliberative quality was operationalized through the discursive features *constructive tone*, *responds to previous comment*, *acknowledges problem*, *topic shift*, *asks question(s)*, *clarifies position*, *provides information*, *gives reason(s)*, and *presents evidence*, which we used as independent variables. The *number of ideas*, as defined in the coding scheme (see Table 3 in section 3.2.1), was the dependent variable. The goal was to examine the relationship between the independent and dependent variables, an established application of regression models (Bruce et al. 2020). We checked for multicollinearity among the independent variables to ensure that correlated variables were not included together in the model. Multicollinearity complicates the correct estimation of the relationship between each independent variable and the dependent variable (Bruce et al. 2020). Similar to Burke et al. (2009); Gil de Zúñiga et al. (2016), and Štětka and Mazák (2014), variance inflation factors (VIFs) were computed to detect multicollinearity in the set of independent variables. The VIF analysis indicated that the variables *constructive tone* and *responds to previous comment* had high multicollinearity. It means that these variables are highly correlated with the other independent variables of the model but not necessarily with each other. Therefore, we decided to remove them from the set of independent variables before running the analysis. The results of this multivariate regression are presented in section 4.1.3.

In the model addressing RQ3, we used *disagreement* and *agreement* as independent variables and *number of ideas* as a dependent variable. Elaboration was included in the model in two ways. First, it was operationalized as a type of dis/agreement: each comment featuring agreement or disagreement was categorized as either simple or elaborated according to the coding scheme (see Table 3 in section 3.2.1). We therefore included the independent variables *simple agreement*, *simple disagreement*, *elaborated agreement*, and *elaborated disagreement* in the regression model. The variable *number of ideas* was used as the dependent variable. We checked for multicollinearity among the independent variables. All VIFs were below 5, indicating the nonexistence of linear dependency among the independent variables (Hocking 2013). We present the results of the multivariate regression analysis in section 4.2.1.

**Fig. 2:**
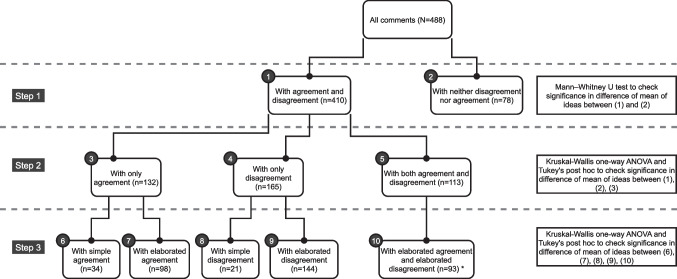
Design of the multi-step inferential analysis (see section 3.2.3) to examine the roles of disagreement and agreement in idea generation in crowdsourced deliberation

#### Multi-Step Inferential Analysis: Disagreement, Agreement, Elaboration, and Idea Generation

To further address RQ3 and examine how the presence/absence of agreement and disagreement and the type of dis/agreement (simple or elaborated) are associated with the number of ideas in crowdsourced deliberation, we complemented the regression analysis with a multi-step inferential analysis. We compared the numbers of ideas in the comments, which were grouped based on the presence of disagreement and/or agreement and the type of dis/agreement (simple or elaborated). The design of this procedure is illustrated in Fig. 2.

In *Step 1*, we divided the 488 comments into two sets: one containing comments in which disagreement and/or agreement occurred and the other containing comments featuring neither agreement nor disagreement. We computed the mean number of ideas in each group. Because the numbers of ideas in the groups of comments did not follow a normal distribution, we used the non-parametric Mann–Whitney U test (Mann and Whitney 1947) to calculate the statistical significance of the difference in the means. *Step 1* enabled us to check (1) whether comments featuring disagreement and/or agreement had on average more ideas than comments without agreement or disagreement and (2) whether the differences were statistically significant. However, this step did not provide enough granularity to distinguish whether a potential difference in the number of ideas might come from comments featuring agreement, comments featuring disagreement, or comments in which both agreement and disagreement were present. Therefore, in *Step 2*, comments featuring disagreement and/or agreement were subdivided into three sets: (1) comments featuring only agreement, (2) comments featuring only disagreement, and (3) comments featuring both agreement and disagreement. As in Step 1, we calculated the mean number of ideas in each group. We used the non-parametric Kruskal-Wallis one-way ANOVA test (Chan and Walmsley 1997) to check whether the differences between the means of the groups of comments was statistically significant. Tukey’s post-hoc test was used to determine which differences between the groups’ means were statistically significant (see Step 2 in Fig. 2).

Based on the results from Step 2, we were able to verify the association between the presence of dis/agreement and the number of ideas. However, we were not able to examine the role of the type of dis/agreement (simple or elaborated). Therefore, we took a third and final step to examine the roles of simple and elaborated dis/agreement in idea generation. In *Step 3*, we divided the comments featuring only agreement into two subsets: one comprising comments that featured only simple agreement and another comprising comments that featured only elaborated agreement. Similarly, the comments featuring only disagreement were separated into two subsets: one comprising comments that featured only simple disagreement and the other comprising comments that featured only elaborated disagreement. The comments in which agreement and disagreement co-occurred were divided into four subsets, each containing one possible combination of simple/elaborated dis/agreement: i) simple agreement and simple disagreement, ii) simple agreement and elaborated disagreement, iii) elaborated agreement and simple disagreement, and iv) elaborated agreement and elaborated disagreement. As in Step 2, the mean number of ideas in each group was computed, and the Kruskal-Wallis one-way ANOVA and Tukey’s post hoc tests were used to test the statistical significance of the differences between the means (see Step 3 in Fig. 2). We report the results of this inferential analysis in section 4.2.2.

#### Participant Survey

To address RQ1—regarding deliberative quality—we distributed an online survey to the registered participants on the platform. 52 of the participants completed the survey, resulting in a 30% response rate. This response rate is consistent with online survey response rates in general Shih and Fan (2008) and similar to survey response rates in prior studies (Aitamurto et al. 2017; Aitamurto and Saldivar 2017b; Aitamurto and Landemore 2016). By responding to the survey, the participants were entered into a raffle for a gift card. The survey inquired about the participants’ demographic profile, experience with association activity, interest in the Association Act, and experience during the crowdsourced deliberation; as well as their perceptions of the roles and value of disagreement in the process, the quality of the deliberation, knowledge and learning during the deliberation, and the value of the crowdsourced information and ideas.

To address RQ1 about deliberative quality, we measured the quality of the crowdsourced deliberation using an 11-item scale adapted from Gutmann and Thompson (1996) and Fishkin (2009) and included in the participant survey (α = .94). The scale included items such as the following: “Interaction in the online discussion was on the whole appropriate and respectful towards other participants”; “Participants genuinely tried to listen to all parties and understand their views”; “For the most part, participants reacted constructively to differing points of view.” All the items are listed in Table 4.

The participants rated the items on a seven-point Likert scale, in which one represented “strongly disagree” and seven represented “strongly agree.” We report the results in section 4.1.1. To address RQ3, we analyzed participants’ responses to open-ended survey questions about their perceptions of deliberative quality, dis/agreement, and elaboration in the crowdsourced deliberation. We analyzed the answers using Strauss and Corbin’s open coding method (Strauss and Corbin 1998). We report the results in section 4.2.3.

**Table 4:** Means, medians, and standard deviations for the deliberative quality scale items in the participant survey

*Statement*	*M*	*Mdn*	*SD*
Interaction in the online discussion was on the whole appropriate and respectful towards other participants.	5.79	6	0.91
Information shared in the discussion was for the most part accurate.	5.29	5	0.9
Participants genuinely tried to listen to all parties and understand their views.	5.24	5	0.88
Participants seemed sincere in their opinions and justifications.	5.58	6	1.03
Participants were trying to come to an understanding.	4.89	5	0.92
Different points of view complemented each other in the online discussion.	5.26	5	1
Disagreements in the online discussion were for the most part constructive.	5.24	5.5	0.94
For the most part, participants reacted constructively to differing points of view.	5.17	5	0.95
For the most part, participants accepted disagreements.	5.2	5	0.87
For the most part, participants in the discussion justified their opinions well.	4.97	5	1.15
The group of participants was diverse and represented well the groups affected by the law.	4.43	4	1.17

#### Participant Interviews

To address RQ1 about deliberative quality, we interviewed 17 participants in the crowdsourced deliberation using a semi-structured interview outline, which inquired about the participants’ experience during crowdsourcing, their interest in association activity and the Association Act, and their perceptions of the quality of deliberation and the roles of disagreement and elaboration in the crowdsourced deliberation. The interviewees were recruited by email through the online platform. A random sample of the participants were contacted for interviews. The interviews were conducted by phone, and the average duration was 30 minutes. The interviewees were active participants in and/or staff members of an association. Eleven of the interviewees were male, and six were female. Their ages ranged from 25 to 69 years old. All interviewees worked full-time, apart from two interviewees who were retired. The interviews were recorded and transcribed. We analyzed the interviews using Strauss and Corbin’s (1998) open coding method, identifying the main themes in the participants’ perspectives on deliberative quality, disagreement, and elaboration in the interviews, as reported in sections 4.1.1 and 4.2.3.

#### Data and Code Transparency

We published the code used in the analysis, the anonymized comment data, and the anonymized survey data in the Open Science Foundation archive.^2^ The link will be made public upon publication of this article.

## Results

In this section, we present the results organized by research question. We first review the results pertaining to RQ1 about deliberative quality in crowdsourced deliberation (section 4.1); followed by RQ2 about the role of deliberative quality in idea generation (section 4.1); and finally RQ3 about the role of disagreement, agreement, and elaboration in idea generation (section 4.2).

### Deliberative Quality and Idea Generation in the Crowdsourced Deliberation

In this section, we report the results pertaining to RQ1 and RQ2 about deliberative quality and its role in idea generation in the crowdsourced deliberation. We first address the question of the deliberative quality of the crowdsourced deliberation using the survey, interview, and online comment data. We then review the results regarding the association between deliberative quality and new idea generation.

#### Participants’ Perspectives on Deliberative Quality

To examine the quality of the crowdsourced deliberation, we analyzed the participants’ answers to the 11 deliberative quality scale statements in the survey (see section 3.2.4). The results show that the participants perceived the quality of deliberation as high (*M* = 5.18, *Mdn* = 5, *SD* = 0.97 on a seven-point Likert scale), as detailed in Table 4. The participants perceived that interactions during the deliberation were appropriate and respectful and that participants were trying to understand each other’s perspective. They also perceived that participants showed genuine interest in others’ opinions, tried to listen to all parties and understand their views, were sincere in their opinions and justifications, reacted constructively to different opinions, and justified their opinions well, all of which are indicators of high deliberative quality (Fishkin 2009; Friess and Eilders 2015; Gutmann and Thompson 1996).

The interview and open-ended survey question analysis also showed that the participants characterized the tone of the crowdsourced deliberation as constructive, civil, and respectful. We refer to the interviewees and survey respondents with a letter and number combination: I1–I17 and S1–S52, respectively. As one interviewee (I6, female) said, “It was a safe place to propose ideas, listen to others, and learn.” Another interviewee (I7, female) said, “The quality of the discussion gave me a feeling that we live in a real democracy, where we can discuss issues and disagree in a constructive manner.” The participants described the tone of the discussion in the crowdsourcing process as constructive, civil, and respectful. Participants shared information with each other throughout the deliberations. The interactions also motivated some participants to extend their information search beyond the platform: “I learned to see the issues from another perspective. One’s own knowledge is often incorrect. After participating, I also went ahead and searched for more information online” (S13, female). These results align with the comment data analysis results we present in the next section, which show that the crowdsourced deliberation had a constructive tone and that the participants generally justified their positions.

#### Deliberative Quality of the Online Comments

To further address RQ1 about the quality of the crowdsourced deliberation, we analyzed the frequencies of discursive features that indicate deliberative quality in the online comment data: *constructive tone*, *responds to previous comment*, *acknowledges problem*, *topic shift*, *asks question(s)*, *clarifies position*, *provides information*, *gives reason(s)*, and *presents evidence*. Table 5 shows the frequency of each feature. In the majority (71%) of comments, the participants justified their stances by giving reasons. The participants thus provided each other with material on which to base rational and constructive disagreement and/or agreement. In almost half of the comments (46%), the participants explicitly acknowledged the existence and/or validity of a problem mentioned in another comment. This result shows that in almost half of the comments, the participants engaged with each other’s ideas and perspectives. This also indicates that the participants did not dismiss each other’s ideas, concerns, and criticisms, which reflects mutual respect and listening, important aspects of deliberative quality (Fishkin 2009; Gutmann and Thompson 1996).

Asking questions (20%), clarifying a position (12%), presenting evidence (8%), and providing information (5%) were less commonly occurring discursive features. Thus, the participants justified their claims by providing reasons but did not provide evidence in the sense of external sources or materials, such as books, newspapers, or government reports. Instead, the participants shared first-hand experiences with the areas governed by the association act by sharing experiental knowledge, i.e. understanding gained through direct personal experience and action (Fazey et al. 2006). The low frequency of the feature “provides information” reflects that participants almost always adopted a stance in their comments (i.e., they expressed disagreement or agreement) rather than providing information in a neutral or nonpartisan manner.

In the vast majority of the comments, the tone was constructive (97%), and there was a high degree of interactivity in the deliberation: A majority (77%) of the comments responded to previous comments, and less than a quarter (24%) of the comments included a topic shift. These results indicate that the discussion was cohesive, participants showed respect towards each other, and there was a high level of reciprocity and engagement in the deliberation. Moreover, the relatively low occurrence of topic shifts in the crowdsourced deliberation may reflect that participants tended to give sustained focus to one category or topic at a time.

**Table 5:** Frequencies of discursive features indicating deliberative quality in the crowdsourced comments

*Coding category*	*No. of comments*	*% of total*
Constructive tone	473	96.93
Responds to previous comment	376	77.05
Acknowledges problem	223	45.70
Topic shift	117	23.98
Asks question(s)	95	19.47
Clarifies position/stance	62	12.70
Provides information	23	4.71
Gives reason(s)	320	78.05$^{1}$
Presents evidence	37	9.02$^{1}$

$^{1}$ The codes “gives reason(s)” and “presents evidence” could only be applied to comments that featured disagreement, agreement, or both, so they are given as percentages of that subset of comments rather than all comments

#### Deliberative Quality and Idea Generation

To address the role of deliberative quality and idea generation in crowdsourced deliberation in RQ2, we report the results of the multivariate linear regression analysis. The results show that the discursive features *acknowledges problem*, *topic shift*, *asks question(s)*, *clarifies position/stance*, and *gives reason(s)* had a statistically significant positive association with the number of ideas, as Table 6 shows. These results suggest that the many of the key features of deliberative quality may indeed contribute to ideation in crowdsourced deliberation. In contrast, the features *presents evidence* and *provides information* did not have a statistically significant association with the number of ideas.

Interestingly, the number of ideas had a positive association with *topic shifts.* In deliberation, frequent topic shifts may reflect that participants are talking past each other rather than listening to and engaging with each other. This would signal a lack of reciprocity and mutual respect and would therefore detract from deliberative quality according to deliberative democratic theory (Bohman 1996; Habermas 1996; Cohen 1989; Gutmann and Thompson 1996). However, this result suggests that in crowdsourced deliberation, topic shifts can contribute to idea generation if they occur infrequently and are timed appropriately. Topic shifts in crowdsourced deliberation can signal expansion of perspectives, which can lead to brainstorming and generation of new ideas, and thus serve as a positive discursive feature rather than a negative one.

**Table 6:** Multivariate linear regression model describing the relationship between new idea generation and discursive features indicating deliberative quality

*Variable*	β	*SE*	*t*	*p*
(Intercept)	0.49	0.09	5.52	0.000$$^{***}$$
Acknowledges problem	0.35	0.08	4.20	0.000$$^{***}$$
Topic shift	0.31	0.09	3.32	0.001$$^{**}$$
Asks question(s)	0.55	0.10	5.27	0.000$$^{***}$$
Clarifies position	0.26	0.12	2.09	0.04$^{*}$
Provides information	0.01	0.19	0.07	0.94
Gives reasons	0.92	0.10	9.47	0.000$$^{***}$$
Presents evidence	-0.03	0.15	-0.19	0.85

$$^{*}$$ *p* < 0.05, $$^{**}$$ *p* < 0.01, $$^{***}$$ *p* < 0.001, N=488, $$R^2=0.27$$

### Dis/agreement, Elaboration, and Idea Generation in Crowdsourced Comments

In this section, we report the results pertaining to RQ3 about the roles of disagreement, agreement, and elaboration in idea generation in crowdsourced deliberation. We first report the results of the multivariate regression analysis and then the results of the multi-step inferential analysis comparing groups of comments, as described in section 3.2.2.

#### Multivariate Regression Analysis Results

Table 7 shows the frequencies of disagreement, agreement, and elaboration in the crowdsourced deliberation. To address RQ3 about the roles of disagreement, agreement, and elaboration in crowdsourced deliberation, we constructed a multivariate linear regression model (Table 8) using the comment analysis codes *simple agreement*, *elaborated agreement*, *simple disagreement*, and *elaborated disagreement*, as described in section 3.2.2. The results suggest that the presence of new ideas in the comments was positively and statistically significantly associated with *elaborated agreement* and *elaborated disagreement*, as Table 8 shows. Discussions in which elaborated agreement or elaborated disagreement was present tended to produce 0.5 more new ideas than comments that did not feature elaborated agreement or elaborated disagreement. Interestingly, simple agreement had a statistically significant but negative association with the presence of ideas in the comments. This factor was negatively correlated with the dependent variable *new ideas* with a magnitude of -0.19, as shown in Table 8.

**Table 7:** Frequencies of the types of disagreement, agreement, and elaboration in online comments

*Coding category*	*No. of comments*	*% of total*	*% within category*$^{1}$
Disagreement	278	56.97	
– Simple disagreement	26	5.33	9.35
– Elaborated disagreement	252	51.64	90.65
Agreement	246	50.41	
– Simple agreement	54	11.07	21.95
– Elaborated agreement	192	39.34	78.05

**Note.** The percentages for “disagreement” and “agreement” do not sum to 100 because both of these codes could be applied to a single comment.

$^{1}$The codes “simple” and “elaborated,” which are mutually exclusive, could only be applied to comments featuring disagreement and/or agreement, so these are presented as percentages of their respective category (e.g., elaborated disagreement is given as a percentage of comments featuring disagreement)

**Table 8:** Multivariate linear regression model for analyzing the association between new idea generation and the types of dis/agreement (simple/elaborated)

*Variable*	β	*SE*	*t*	*p*
(Intercept)	1.13	0.08	13.58	0.000$$^{***}$$
Simple agreement	-0.51	0.14	-3.63	0.000$$^{**}$$
Elaborated agreement	0.50	0.10	5.50	0.000$$^{**}$$
Simple disagreement	-0.19	0.20	-0.96	0.34
Elaborated disagreement	0.50	0.10	5.63	0.000$$^{**}$$

$$^{**}$$ *p* < 0.01, $$^{***}$$ *p* < 0.001, N=488, $$R^2=0.18$$

#### Multi-Step Inferential Analysis Results

To further examine RQ3, we analyzed the association between simple and elaborated dis/agreement and idea generation by applying multi-step inferential analysis, in which we compared crowdsourced comment groups through a three-step process as described in section 3.2.2. We present the results in the following.

As shown in Table 9, 410 of the 488 comments (84%) featured agreement and/or disagreement. These comments produced on average 1.56 ideas. On the other hand, comments with neither agreement nor disagreement (*n* = 78) generated on average 1.17 ideas. Step 1 of the analysis showed that the difference between these means was statistically significant (p<0.001) based on the Mann–Whitney U test. This result indicates that comments containing agreement and/or disagreement featured, on average, a statistically significantly greater number of ideas than comments without disagreement or agreement.

**Table 9:** Numbers of comments and means of the number of ideas in crowdsourced comments grouped according to the featured type(s) of dis/agreement (simple/elaborated)

	*Comment group*	*n*	*M (no. of ideas)*
**Step 1**			
1	Agreement and/or disagreement	410	1.56
2	Neither agreement nor disagreement	78	1.17
**Step 2**			
3	Only agreement	132	1.36
4	Only disagreement	165	1.52
5	Both agreement and disagreement	113	1.97
**Step 3**			
6	Only simple agreement	34	0.35
7	Only elaborated agreement	98	1.70
8	Only simple disagreement	21	0.76
9	Only elaborated disagreement	144	1.63
10	Elaborated agreement and elaborated disagreement	93	2.07
11	Simple agreement and simple disagreement	5	1.20
12	Simple agreement and elaborated disagreement	15	1.47
13	Elaborated agreement and simple disagreement	0	—

In Step 2, we measured the differences between the mean numbers of ideas in the groups of comments with only agreement, only disagreement, and both agreement and disagreement (see comment groups 3, 4, and 5 in Table 9). The Kruskal-Wallis one-way ANOVA showed that the differences between the means of the comment groups were statistically significant (p<0.001). A Tukey’s post-hoc analysis of these groups showed that comments that featured both disagreement and agreement contained a statistically significantly greater number of ideas than comments with only agreement (p<0.01) and comments with only disagreement (p<0.05).

In Step 3, we tested the differences between the mean numbers of ideas of comment groups with only simple agreement, only elaborated agreement, only simple disagreement, only elaborated disagreement, and both elaborated agreement and elaborated disagreement (see comment groups 6, 7, 8, 9, and 10 in Table 9). As outlined in Table 9, the comment groups featuring simple agreement and simple disagreement (*n* = 11), simple agreement and elaborated disagreement (*n* = 12), and elaborated agreement and simple disagreement (*n* = 13) were not large enough to be included in the analysis. The Kruskal-Wallis one-way ANOVA indicated that the remaining groups of comments (6, 7, 8, 9, and 10) differed statistically significantly in the average numbers of ideas they featured (p<0.001).

A post-hoc analysis with Tukey’s test showed that comments featuring both elaborated agreement and elaborated disagreement produced on average a statistically significantly greater number of ideas than comments featuring simple disagreement and simple agreement (p<0.01 and p<0.01, respectively) and comments featuring only elaborated disagreement (p<0.01). Tukey’s test also showed that comments featuring elaborated agreement had a statistically significantly greater number of ideas than comments featuring simple disagreement and simple agreement (p<0.01 and p<0.01, respectively). Similarly, the post-hoc test indicated that comments featuring elaborated disagreement contained a statistically significantly greater number of ideas than comments featuring simple disagreement and agreement (p<0.01 and p<0.01, respectively).

Taken together, these results indicate that 1) comments featuring disagreement and agreement contained more ideas than comments without disagreement or agreement; 2) comments featuring elaborated disagreement and elaborated agreement had more ideas than comments with only simple disagreement or simple agreement; and 3) comments featuring *both* elaborated disagreement *and* elaborated agreement contained more ideas than comments featuring only simple disagreement or simple agreement.

#### Participants’ Perceptions of Elaboration and Disagreement in Crowdsourced Deliberation

To further address RQ3, and particularly the roles of disagreement and elaboration in crowdsourced deliberation, we analyzed the interviews and the open-ended survey responses. Using Strauss and Corbin’s (1998) open coding method, we identified four main themes that describe the participants’ perspectives on deliberative quality, disagreement, and elaboration: *broadening the scope*, *challenging one’s stance*, *new ideas and compromises*, and *downsides of disagreement*. In the following, we describe these categories. We refer to the interviewees and survey respondents with a letter and number combination (I1–I17 and S1–S52, respectively).

**Broadening the scope**. The participants said that elaborated disagreement in the crowdsourced deliberation helped them recognize and better understand others’ perspectives—“leaving one’s bubble,” as one participant (S27, male) put it. This broadened the scope of the policy discussion and helped participants identify the most important factors to consider when evaluating policy proposals, as the participants described:

“Any issue with the policy could be interpreted one way, or another way. Disagreement made diverse perspectives visible on the platform” (S5, female).

“There’s no development, if everybody always agrees – nothing changes. And you can develop your own perspective by reading others’. There’s new perspectives that I didn’t know to consider before” (S37, female).

Disagreement also gave participants opportunities to learn about diverse experiences and practical issues related to the deliberation. As one interviewee described, “I realized that there is a huge variety of association sizes, which creates different needs for the policy” (I9, male). The deliberation gave participants access to each other’s viewpoint, which revealed fundamental differences in their circumstances. For instance, one participant described how reading about other participants’ experiences of working in small associations helped them understand the differences between the operations of smaller and larger associations: “It is helpful to have all perspectives surface because it’s easy to become blind to your own work after having worked a long time in an association” (S48, female).

**Challenging one’s stance**. Encountering elaborated disagreement challenged the participants to reconsider their own stances. As one participant said, “It made me question my own opinion, and I ended up revising it” (I3, male). On the other hand, some interviewees described experiencing a contrary effect when they encountered differing opinions: “Reading their justifications made me feel stronger about my own stance” (I16, male). Thus, encountering elaborated disagreement challenged participants’ stances, leading them to either modify their stances (opinion change) or strengthen their conviction (no opinion change). The deliberations also led the participants to question the status quo, the current state of the law, and the policy proposals introduced by the facilitators and other participants. As one participant (I5, male) described, “It was illuminating to see diverse viewpoints and debate between them, it helped me understand that the existing law and solutions are not necessarily the only ways to go.” In the participants’ accounts, elaboration of viewpoints was closely connected to constructive disagreement. The interviewees and survey respondents emphasized the importance of well-justified, sufficiently elaborated stances for the benefits of disagreement to emerge:

“It was the constructive tone and sincerely good intentions in the participants’ comments that helped me see value in opposing viewpoints” (I11, female).

“When the focus was on identifying solutions, not yanking or proving a point, disagreement was productive” (I4, male).

“Elaborated justifications for diverse viewpoints introduced new information and new perspectives” (I17, male).

**New ideas and compromises**. The interviewees perceived elaborated disagreement as conducive to new idea generation and compromises. They perceived that elaborated, well-justified arguments expressing disagreement contributed to the emergence of diverse perspectives and served as a catalyst for new ideas: “There were new solutions that sprouted from debate about a proposed idea” (I5, male). Expanding the scope of the deliberation helped participants identify new solutions. For instance, a participant (I3, female) described an instance in which she “heard the first fully-fledged counter argument about the CEO role in associations.” “The argumentation around that produced a new solution: a restriction to the CEO role,” she said. While exchanging arguments, participants reconciled previous ideas with new information to develop compromises. In some cases, the compromise or newly identified solution was the result of opinion change: “[Disagreement] provoked completely new thinking models, which I developed further during the online discussion, and that led for changing my opinion. Without disagreement there is not innovation and development, in policy-making either” (S12, female).

**Downsides of disagreement**. The participants also acknowledged the downsides of disagreement. While elaborated disagreement can lead to idea generation and expand the scope of the deliberation, it can also lead to tangents, as an interviewee described (I5, male): “At some points the debate about some details led to side tracks, and it was hard to see the big picture after that.” Too much divergence could thus result in a loss of focus in the deliberation. Moreover, while participants can benefit from exposure to diverse viewpoints as they challenge and problematize each other’s perspective, this can also lead to counterproductive disagreement. The interviewees and survey respondents perceived a lack of elaboration in the comments as unproductive because it led to entrenched disagreements: “If the participants are not listening to each other, problems may occur; in this online discussion I didn’t experience much of that; there was disagreement but the opinions were well justified” (S8, male). The interviewees and survey respondents perceived that unconstructive disagreement could stall the deliberative process. By unconstructive disagreement, the interviewees meant disagreement that lacks elaboration, focuses on trivial details, or stubbornly adheres to a stance without acknowledging other perspectives—qualities that represent the opposite of civil disagreement (Black and Wiederhold 2014; Friess and Eilders 2015). In such instances, disagreement could contribute to a negative atmosphere and polarization. One respondent described unconstructive disagreement thus: “[Disagreement is harmful] only if it leads to nasty back-and forth and ‘hedgehog’ positions, which didn’t happen much here” (S22, male). The respondents perceived asymmetrical knowledge levels as one reason for unconstructive disagreement: “Sometimes I wondered if we were talking about the same topic. Discrepancy in the levels of knowledge can lead to talking past each other” (S33, female).

## Limitations

The main limitation of this study is that it is based on one crowdsourced deliberation process. Having several crowdsourced deliberation processes in the sample would diversify and expand the scope of the policy issues under consideration, which could affect the kinds of discursive features and idea generation we would observe.However, simultaneously studying several crowdsourced deliberation cases, which take place in the wild, is not a trivial challenge from a data gathering perspective. Many empirical studies about crowdsourced policymaking are based on one case (e.g., Aitamurto and Landemore 2016; Aitamurto and Saldivar 2017b; Aitamurto et al. 2017; No et al. 2017; Menendez-Blanco and Bjørn 2022; Chen and Aitamurto 2019; Kim et al. 2019), which has been identified as a valuable approach in recent scholarship on crowdsourcing (Gellers 2016). Moreover, despite the limited scope of a single crowdsourcing process in this study, we believe that the results make a valuable contribution to the body of knowledge about crowdsourced deliberation and other processes that include similar elements, such as crowd participation, idea generation, and deliberation, which are present in several civic technologies (Johnson et al. 2020; Kim et al. 2021; Menendez-Blanco and Bjørn 2022; Saldivar et al. 2019).

Another limitation is the self-selected nature of crowdsourcing as a participatory democracy method. It is a self-selected group who elect to participate in crowdsourced deliberation, so their perspectives and experiences do not necessarily represent those of the general public, as we discussed in section 2.1. Moreover, using a survey sampling method based on self-selection introduces another challenge. The survey results are based on responses from those participants who a) chose to participate in the crowdsourced deliberation and b) chose to respond to the survey. This double self-selection can potentially introduce biases based on the respondent profile and limits the generalizability of the findings. However, these limitations are present in most crowdsourcing processes due to the self-selection inherent in crowdsourcing.

## Discussion

### Deliberative Quality and Ideation in Crowdsourced Deliberation

The findings show that the deliberative quality (RQ1) of this crowdsourced deliberation was high according to both the participants’ perceptions and the deliberative quality of the online comments. The tone of the interactions was constructive, and the participants justified their stances and showed respect and reciprocity towards each other, all of which are positive indicators of deliberative quality (Black and Wiederhold 2014; Gutmann and Thompson 1996; Fishkin 2009; Stromer-Galley 2007). This was despite the lack of moderation, in-person interactions, and shared evidence and information for the basis of the deliberation, factors that are hallmarks of conventional democratic deliberation settings (Fishkin 2009; Gutmann and Thompson 1996; Habermas 1996; Mansbridge 2010). These findings suggest that crowdsourced deliberation can indeed achieve high deliberative quality despite its differences from more traditional forms of deliberation. The findings also signal that crowdsourced deliberation can, at least in this case, contribute to a more democratic society by providing spaces for high-quality deliberation with constructive argumentation.

Furthermore, the results show that the number of ideas in the comments was positively associated with discursive features (RQ2) that indicate engagement and reciprocity and thus are key indicators of deliberative quality, including acknowledging the legitimacy or validity of a problem pointed out by another participant, asking genuine questions, clarifying a position, and giving reasons to support claims and ideas. These findings provide empirical support for the theorized potential of crowdsourced deliberation to generate new ideas (Landemore and Page 2015; Aitamurto and Landemore 2016; Landemore 2020). But more importantly, these findings signal that deliberative quality can contribute to idea generation in crowdsourced deliberation. Deliberative quality can thus contribute to both of the goals of crowdsourced deliberation: constructive argumentation and ideation. Considering this finding, we recommend that civic technologies and participatory democracy applications incorporate features that foster high-quality deliberation because these in turn would also foster ideation.

Interestingly, the number of ideas also had a positive association with *topic shifts*. In more conventional deliberation settings, frequent topic shifts may reflect that participants are talking past each other rather than listening to and engaging with each other (Stromer-Galley 2007; Gutmann and Thompson 1996). This would indicate a lack of reciprocity and mutual respect and would therefore detract from deliberative quality (Bohman 1996; Habermas 1996; Cohen 1989). However, the positive association between topic shifts and the number of ideas may signal that in crowdsourced deliberation, topic shifts can contribute to idea generation without reducing deliberative quality. Topic shifts in a crowdsourced deliberation may expand its diversity and scope and redirect participants’ attention and energy if they have exhausted their potential to deliberate and generate ideas within a particular topic, consistent with findings from research on collaborative creativity processes (Paulus et al. 2019). This in turn can initiate a new round of brainstorming and thus generate new ideas, a key goal in crowdsourced deliberation.

In contrast, the discursive features *presents evidence* or *provides information* did not have a positive association with new idea generation. These features, however, have been identified as fundamentally important factors for reasoned and productive disagreement and deliberation (Stromer-Galley 2007). This difference may have been due to the context of this crowdsourced deliberation: Instead of presenting empirically verifiable evidence—such as shared documents, news articles, or government reports, as in more conventional forms of deliberation—participants shared information based on their individual experiences with and knowledge of associations to support their arguments, i.e., experiential knowledge, which means understanding gained through direct personal experience and action. However, even without the use of external evidence and information, the participants were able to engage in high-quality deliberation and generate new ideas.

The absence of external evidence in the comments may stem from multiple factors. It may reflect participants’ trust in one another, confidence in their own knowledge, and the non-controversial nature of the deliberation as a whole. When the environment is civil and participants’ views are not challenged, there can be less perceived need to substantiate claims with external evidence. While experiential knowledge can enrich policy deliberations by grounding abstract discussions in lived realities and firsthand experiences—thereby humanizing complex issues and strengthening the democratic legitimacy of deliberative processes—it also presents challenges in crowdsourced deliberation. Individual experiences are often context-specific and may not represent broader populations. Therefore, in crowdsourced deliberation, experiential knowledge must be interpreted cautiously to avoid drawing conclusions that are overly narrow or anecdotal. Future research should examine how to achieve a meaningful balance between personal experience and evidence-based information sharing in crowdsourced deliberation.

These findings suggest how the dynamics in crowdsourced deliberation may differ from more traditional deliberations. Future work should examine the roles of topic shifts and providing evidence or information in crowdsourced deliberation, especially their impact on ideation.

### Elaborated Dis/agreement and Ideation in Crowdsourced Deliberation

We also found that the simultaneous presence of disagreement and agreement in comments was more strongly positively associated with idea generation (RQ3) than the presence of either disagreement or agreement alone or the absence of both. Moreover, the simultaneous presence of elaborated disagreement and elaborated agreement had the strongest positive association with idea generation. This suggests that elaboration (i.e., justifying or further explaining a claim or proposal)—already considered an important marker of deliberative quality (Stromer-Galley 2007)—can also contribute to idea generation, a key goal of crowdsourced deliberation. The survey and interview responses also indicated the importance of both disagreement and elaboration as essential elements of deliberation that also foster idea generation. This finding aligns with prior work showing that sharing rationales in online ideation tasks improves the number and quality of generated ideas (Xiao 2014, 2011, 2013).

The results suggest that through constructive argumentation, crowdsourced deliberation can contribute to an informed and engaged citizenry while also producing ideas for improving policy. Moreover, crowdsourcing can enable deliberation and idea generation to take place at a larger scale and at lower cost than traditional forms of face-to-face deliberation. The results also indicate that elaboration, disagreement, and agreement are intertwined features in deliberation and should be approached as such in research as well as process and platform design. Furthermore, these results emphasize the importance of elaboration in conjunction with dis/agreement in crowdsourced deliberation. The design of processes and technologies for crowdsourced deliberation should foster both elaborated disagreement and elaborated agreement to maximize both deliberative quality and idea generation. To this end, deliberation and ideation processes and platforms should incorporate features from both large-scale ideation technologies *and* argumentation tools to support *both* idea generation *and* constructive deliberation characterized by the presence of both elaborated disagreement and elaborated agreement. To advance these goals, we present design considerations and directions for future work in section 6.3.

### Design Considerations and Future Work

Based on the findings described in the previous sections, we present considerations for the design of technologies and processes for civic technologies, which facilitate crowdsourced deliberation. Elaboration of perspectives accompanying both disagreement and agreement was a key factor in contributing to productive ideation. Therefore, the design of processes and platforms for crowdsourced deliberation should prioritize features that encourage elaboration of perspectives in deliberation. Here we suggest some examples of such features as well as existing platforms and tools that incorporate them in some form.

First, showing examples of well justified comments could nudge participants to elaborate their viewpoints. Platforms for crowdsourced deliberation could provide functions for moderators and participants to choose and display well justified arguments as “featured comments,” similar to SolutionChat (Lee et al. 2020). Second, because elaboration generally involves participants justifying their perspectives, a short comment may indicate a lack of elaboration. Therefore, participants could be prompted through manual and/or automatic notifications to extend their rationales if they submit short comments (e.g., below a specified word count), similar to nudging features described by Menon et al. (2020). In addition, chatbots could be used as automated moderators (Kim et al. 2021) or to assist human moderators (Lee et al. 2020) to elicit elaboration from the discussants. Third, to help participants follow the exchange of arguments and better formulate their own rationales, deliberation platforms could incorporate features such as an argument mapping structure, similar to Consider.it (Kriplean et al. 2012), which enables participants to express and view the pros and cons of proposals by highlighting opposing and supportive arguments in a visually clear and engaging manner.

This study is one of the first steps towards developing a greater understanding of crowdsourced deliberation. Future research should further examine the relationship between discursive features of crowdsourced deliberation and idea generation. It would be particularly valuable to examine the association between certain discursive features and ideation in controlled experiments. Furthermore, dividing instances of elaboration into more nuanced categories and analyzing the effects of different types of elaboration could provide valuable knowledge to further our understanding of the role of elaboration in crowdsourced deliberation. Another important research direction is to examine the quality of ideas and the relationship between idea quality and markers of deliberative quality.

In this study, we examined factors that affect deliberative quality in crowdsourced deliberation at the discourse level. However, several process-level factors may also influence deliberation, including cultural habits of interpersonal communication, the structured nature of crowdsourced deliberation compared to free-form online discussions, the moderator’s role, and the topic of deliberation. While examining these factors were beyond the scope of this study, they are highly relevant to crowdsourced deliberation and should be explored in future research. For example, the topic of deliberation in this case—the reform of an association act—was arguably less controversial than subjects like immigration or other social policies, which may have influenced the tone of the discourse. Future research should, therefore, examine crowdsourced deliberation in the context of different policy areas.

Furthermore, another important consideration for future work is the facilitators’ role. Facilitators’ active participation and visibility on the platform, as well as the constructive tone of their high-quality comments, may have contributed to a more civil and constructive overall deliberation. The facilitators’ presence on the platform may have served as an implicit form of moderation, particularly due to their status as representatives of the Ministry of Justice. Findings from previous research on facilitators’/moderators’ roles in deliberation are inconclusive. On the one hand, scholars suggest that active facilitation can lead to democratic and discursive benefits (Wright 2009), promote polite behavior and high-quality reasoning (Zhang et al. 2013), and result in a higher inclusion of traditionally excluded communities (Trénel 2009). On the other hand, problems arose when facilitation was poorly designed and organized, leading to allegations of censorship, abusive practices, and counterproductive outcomes (Coleman et al. 2002). Based on the existing literature and given the limitations of the design of our study, we can only speculate about the influence of facilitators’ activities, visibility, and presence. This emphasizes the need for research specifically focused on facilitators’ roles in deliberation. Additionally, the process of evaluating and incorporating crowdsourced input into policy is a critical area that warrants further study.

## Conclusion

We examined the roles of deliberative quality, dis/agreement, and elaboration in crowdsourced deliberation within a crowdsourced policymaking process. The findings show that despite the many features of traditional democratic deliberation setting, e.g., the lack of structured moderation and facilitation, the quality of deliberation was high, and the participants showed respect and reciprocity towards each other. Discursive features indicating deliberative quality and elaborated disagreement and agreement had a positive association with idea generation. The presence of both elaborated disagreement and elaborated agreement was conducive to ideation.

The findings suggest that deliberative quality can contribute to both of the dual goals of crowdsourced deliberation: idea generation and constructive argumentation. The many theorized benefits of democratic deliberation—including the development of new ideas and compromises, perspective-taking, increased understanding, learning, and information exchange—were present in this crowdsourced deliberation despite its differences from more traditional forms of deliberation. The findings thus signal validation for crowdsourced deliberation as a democratic innovation that can indeed foster constructive, high-quality deliberation while also generating a large volume of ideas. Through appropriate design interventions—particularly those that support deliberative quality and elaboration in crowdsourced policy deliberation—and by adopting features from both large-scale ideation and deliberation and argumentation tools, both of these aspects—idea generation and constructive argumentation—can be bolstered.

## Data Availability

Data and Code Transparency: Upon publication of this article, we will publish the code used in the analysis in the Open Science Foundation (1https://osf.io/rjn3x/?view_only=709f08c511704ed5a80f8cf87fb80027).

## Appendix A Comparative Analysis of Participant and Facilitator Comments

To compare participant comments with facilitator comments, we present the numbers and percentages of comments featuring each coding category for participant comments only (Tables 10 and 11) and facilitator comments only (Tables 12 and 13). These data indicate that participants’ and facilitators’ comments exhibited similar distributions of discursive features for the most part. The two sets of comments differed the most in the frequencies of the codes *disagreement*, *agreement*, *elaborated** disagreement*, *simple agreement*, *provides information*, and *responds to previous comment*. These differences are likely due in part to differences in expectations and norms for the participant and facilitator roles as well as the structure and format of the crowdsourced deliberation.

**Table 10:** Comment data analysis: Discursive features associated with deliberative quality (participant comments only)

Coding category	Number of comments	Percentage of total
Discursive Features		
Constructive tone	352	95.91
Responds to previous comment	297	80.93
Acknowledges problem	175	47.68
Topic shift	91	24.80
Asks question(s)	77	20.98
Clarifies position/stance	48	13.08
Provides information	9	2.45
Gives reason(s)	274	84.83*
Presents evidence	27	8.36*

**Notes.**

*The codes “gives reason(s)” and “presents evidence” could only be applied to comments that featured disagreement, agreement, or both, so they are given as percentages of that subset of comments rather than all comments

The participants expressed more disagreement—and more elaborated disagreement in particular—than facilitators. This is likely due in part to the basic structure of the crowdsourced deliberation: facilitators posted the initial versions of proposals developed prior to the crowdsourced deliberation, to which participants were then invited to respond. In other words, the participants were invited to provide feedback on and/or present alternatives to existing proposals, whereas the facilitators mainly presented existing proposals for participants to consider. This basic structure contributed to a higher rate of disagreement in participant comments than in facilitator comments because many participants responded to each initial proposal (presented by a facilitator), often raising objections or pointing out potential problems. Participants also responded to and disagreed with each other, whereas facilitators typically only responded to participants’ comments and not to initial proposals or other facilitators’ comments. In responding to proposals and to each other’s comments, participants tended to give reasons for their stances.

**Table 11:** Comment data analysis: Disagreement, Agreement, and Elaboration (participant comments only)

Coding category	Number of comments	Percentage of total	Percentage within category*
Disagreement	227	61.85	
– Simple disagreement	23	6.27	10.13
– Elaborated disagreement	204	55.59	89.87
Agreement	194	52.86	
– Simple agreement	47	12.81	24.23
– Elaborated agreement	147	40.05	75.77

**Notes.** The percentages for “disagreement” and “agreement” do not sum to 100 because one or both of these codes could be applied to a single comment.

* The codes “simple” and “elaborated,” which are mutually exclusive, could only be applied to comments featuring disagreement and/or agreement, so these are presented as percentages of their respective category (e.g., elaborated disagreement is given as a percentage of comments featuring disagreement)

**Table 12:** Comment data analysis: Discursive features associated with deliberative quality (facilitator comments only)

Coding category	Number of comments	Percentage of total
Discursive Features		
Constructive tone	121	100
Responds to previous comment	79	65.29
Acknowledges problem	48	39.67
Topic shift	26	21.49
Asks question(s)	18	14.88
Clarifies position/stance	48	13.08
Provides information	14	11.57
Gives reason(s)	72	82.76*
Presents evidence	11	12.64*

**Notes.**

*The codes “gives reason(s)” and “presents evidence” could only be applied to comments that featured disagreement, agreement, or both, so they are given as percentages of that subset of comments rather than all comments

Participants expressed disagreement in their responses to proposals introduced by facilitators and to each others’ comments, raising potential issues with proposals and challenging each others’ claims. The facilitators did not challenge or problematize the proposals they introduced, instead leaving this to participants and occasionally presenting alternative proposals either immediately following an initial proposal or after a series of participant comments discussing an initial proposal. Facilitators also occasionally disagreed with participants about matters like the proper interpretation of or factual information about an existing policy. However, the participants, rather than the facilitators, were the main source of comments exhibiting the building function of disagreement.

**Table 13:** Comment data analysis: Disagreement, Agreement, and Elaboration (facilitator comments only)

Coding category	Number of comments	Percentage of total	Percentage within category*
Disagreement	52	42.98	
– Simple disagreement	3	2.48	5.77
– Elaborated disagreement	49	40.50	94.23
Agreement	52	42.98	
– Simple agreement	7	5.79	13.46
– Elaborated agreement	45	37.19	86.54

**Notes.** The percentages for “disagreement” and “agreement” do not sum to 100 because one or both of these codes could be applied to a single comment.

* The codes “simple” and “elaborated,” which are mutually exclusive, could only be applied to comments featuring disagreement and/or agreement, so these are presented as percentages of their respective category (e.g., elaborated disagreement is given as a percentage of comments featuring disagreement)

One of the main goals of the crowdsourced deliberation was to elicit participants’ diverse local knowledge and experiences with associations to improve policy. In response to a proposal or other participants’ comments, participants sometimes expanded the scope of the discussion by introducing a new context, use case, or consideration. Facilitators prompted participants to share such perspectives but generally did not do so themselves, likely due to typical expectations and norms regarding participant and facilitator roles.

The higher frequencies of agreement and simple agreement in participant comments compared with facilitator comments also likely reflect the structure of the crowdsourced deliberation and different expectations and norms for participants and facilitators. Nearly all expressions of simple agreement were participants’ responses to proposals presented by facilitators in which they expressed support for a proposal without elaborating, such as by commenting, “I support this proposal.” The higher rate of agreement in participant comments likely reflects that participants expressed agreement with initial proposals and with each others’ comments, whereas facilitators generally only responded to participants’ comments.

The code *provides information* was applied to comments that presented information in a neutral or nonpartisan manner, i.e., without indicating a particular stance or expressing (dis)agreement. This occurred more often in facilitator comments than participant comments, likely due to different expectations, norms, and sets of knowledge associated with these roles. The facilitators tended to have more precise and extensive knowledge about policy details and factual information pertaining to the deliberation, and facilitators are typically expected to maintain a neutral or nonpartisan stance. This likely explains why the facilitators provided information in a neutral or nonpartisan manner more often than participants.

The participants responded to a previous comment more frequently than facilitators, which again is likely due to typical expectations and norms for these roles and the structure of the crowdsourced deliberation, in which facilitators posted initial proposals and then invited participants to respond. Participants responded to these proposals and to each other, whereas facilitators replied only to participants’ comments.

## Footnotes

1 To avoid the constraints of time, space, and resources, some recent efforts have initiated an online replication of the traditional democratic deliberation model by adopting features that support representative sampling, facilitation, and information provision, focusing particularly on automating the moderation of the deliberation (Fishkin et al. 2019).

2 https://osf.io/rjn3x/?view_only=709f08c511704ed5a80f8cf87fb80027
